# Supplementary material for: Genotypic Diversity of Candida parapsilosis Complex in Invasive Candidiasis at a Pediatric Tertiary Hospital: A 5-Year Retrospective Study
Source: J Fungi (Basel). 2022 Dec 6;8(12):1280. doi: 10.3390/jof8121280 (PMC9784890; doi:10.3390/jof8121280)
Supplement: Supplementary file 1 [file jof-08-01280-s001.zip › jof-2073079-supplementary.pdf]

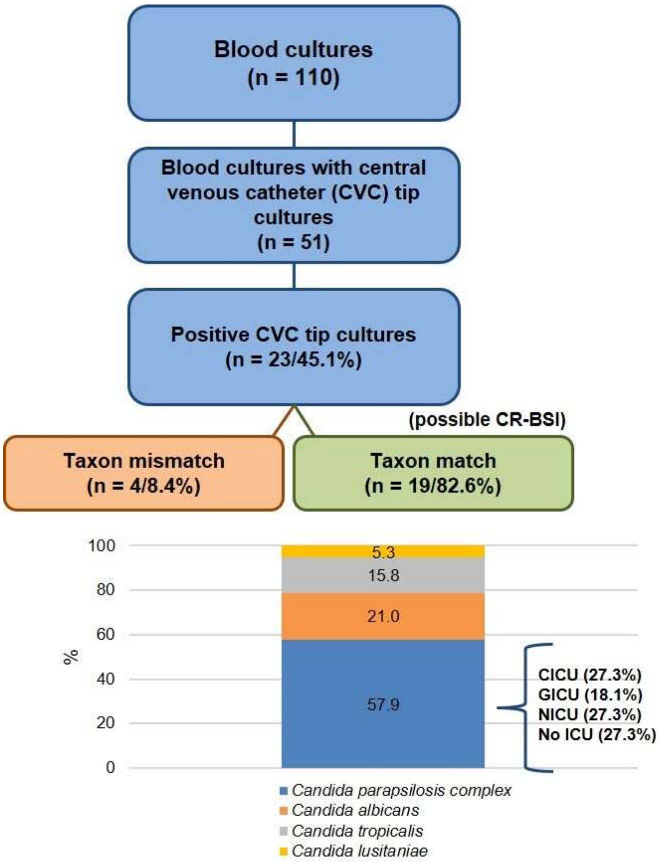

Figure S1. Survey of patients with blood cultures and central venous catheter (CVC) tip cultures among IC patients included in the study. CICU, cardiac intensive care unit; NICU, neonatal intensive care unit; GICU, general intensive care unit; CR-BSI, catheter related bloodstream infection.
